# THE AGING NETWORK AND THE OAA REAUTHORIZATION

**DOI:** 10.1093/geroni/igz038.855

**Published:** 2019-11-08

**Authors:** Amy Gotwals

**Affiliations:** National Association of Area Agencies on Aging, Washington, District of Columbia, United States

## Abstract

Area Agencies on Aging and the aging network are the key service organizers and providers across the nation. This session will address the goals of the aging network for the OAA reauthorization and the trends and innovations in community-based service delivery.

